# Inflammation, It’s Regulation and Antiphlogistic Effect of the Cyanogenic Glycoside Amygdalin

**DOI:** 10.3390/molecules26195972

**Published:** 2021-10-01

**Authors:** Daniela Figurová, Katarína Tokárová, Hana Greifová, Nikola Knížatová, Adriana Kolesárová, Norbert Lukáč

**Affiliations:** Department of Animal Physiology, Faculty of Biotechnology and Food Science, Slovak University of Agriculture in Nitra, Trieda Andreja Hlinku 2, 949 76 Nitra, Slovakia; xfigurova@uniag.sk (D.F.); hana.greifova@uniag.sk (H.G.); xknizatovan@uniag.sk (N.K.); adriana.kolesarova@uniag.sk (A.K.); norbert.lukac@uniag.sk (N.L.)

**Keywords:** amygdalin, cyanide toxicity, inflammation, inflammatory mediators

## Abstract

The inflammatory reaction accompanies in part or in full any disease process in the vascularized metazoan. This complicated reaction is controlled by regulatory mechanisms, some of which produce unpleasant symptomatic manifestations of inflammation. Therefore, there has been an effort to develop selective drugs aimed at removing pain, fever, or swelling. Gradually, however, serious adverse side effects of such inhibitors became apparent. Scientific research has therefore continued to explore new possibilities, including naturally available substances. Amygdalin is a cyanogenic glycoside present, e.g., in bitter almonds. This glycoside has already sparked many discussions among scientists, especially about its anticancer potential and related toxic cyanides. However, toxicity at different doses made it generally unacceptable. Although amygdalin given at the correct oral dose may not lead to poisoning, it has not yet been accurately quantified, as its action is often affected by different intestinal microbial consortia. Its pharmacological activities have been studied, but its effects on the body’s inflammatory response are lacking. This review discusses the chemical structure, toxicity, and current knowledge of the molecular mechanism of amygdalin activity on immune functions, including the anti-inflammatory effect, but also discusses inflammation as such, its mediators with diverse functions, which are usually targeted by drugs.

## 1. Introduction

Over the last two decades, one of the key discoveries in the field of medicine has been the finding that inflammation is not only part of some specific disorders but is partially or completely involved in a comprehensive range of diseases of mental or physical origin, and these health problems are among the most common causes of morbidity and mortality at present [1]. Inflammation is a biological defense response in the vascularized tissues of multicellular organisms to a stimulus such as a foreign pathogen or internal tissue damage [2]. Arachidonic acid, as one of the key regulators of inflammation, can modulate its course through its products of individual enzymatic pathways [3]. Arachidonate metabolites represent a multi-step solution to the cause but also painful concomitant symptoms, depending on the enzymatic pathway from which they originate [4]. Therefore, pharmacological and medical research is increasingly turning its attention to drugs capable of affecting inflammation, especially at the level of metabolites of the symptomatic Cyclooxygenase-2 (COX-2) pathway [5].

The subject of interest of similar studies and this review is the natural cyanogenic product amygdalin. Over the last ten years, research on cyanogenic glycosides has exploded. The cytotoxic effect of amygdalin on cancer cells in vitro and the distribution of amygdalin in plants that are commonly consumed in the human diet are two of the most popular topics of research. This bioactive glycoside naturally occurs in organic sources, e.g., bitter-flavored almonds, peach kernels, apricots, or plums [6,7]. Its presence in kernel seeds is estimated in approximately 800 plants [8]. However, we do not classify it as a modern compound, as it has been used in traditional and alternative medicine for centuries due to its numerous medicinal benefits [9,10]. It has offered help in relieving pain; fever; or suppressing cough, thirst, and nausea, and later as cancer prevention and treatment [7,11].

However, in addition to all the reported effects of amygdalin, its toxicity due to hydrogen cyanide, which is released mainly when encountered with the gastric enzyme β-glucosidase [12], remains a controversial topic in scientific circles and threatens cell viability at certain concentrations [13]. Table 1 shows the metabolites, adverse effects, overdose treatment, and pharmacological function of amygdalin.

## 2. Amygdalin

### 2.1. Chemistry of Amygdalin

**Figure 1:**
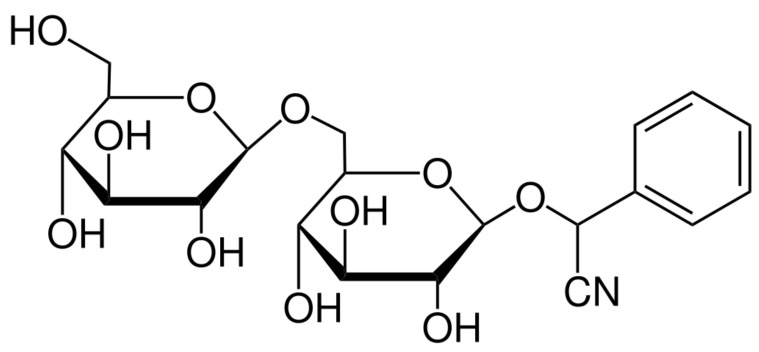
Chemical structure of amygdalin [21].

Amygdalin (AMG) is representative of organic compounds of cyanogenic glycosides with the molecular formula C_20_H_27_NO_11_ (Figure 1) and a molecular weight of 457.42 g.mol^−1^. Amygdalin consists of benzaldehyde, hydrocyanic acid, and two molecules of glucose (d-mandelonitrile-β-d-glucoside-6-β-glucoside) [14]. It was first isolated from bitter-flavored almonds by Robiquet and Boutron-Charlard in the 1930s and was later found to occur naturally as a cyanogenic glycoside in the fruits and seeds of apricots, peaches, bitter-flavored almonds, black cherries, plums, apples, etc. [15,16]. Years of research on the effects of amygdalin have demonstrated its wide range of properties, including its supportive function in the treatment of asthma, bronchitis, leprosy, or colorectal cancer. Amygdalin is also characterized by analgesic effects in which benzaldehyde is an important component [14,17,18]. The anticancer activity of amygdalin is still controversial and has been the subject of much research. This ability may be associated with enzymatic hydrolysis, which leads to the release of hydrocyanic acid [19]. Amygdalin is also identified with the terms “laetrile” and “vitamin B-17”, but both terms are incorrectly used concerning amygdalin [20].

### 2.2. Toxicity of Amygdalin

The metabolism of the drug or any foreign substance entering the body is affected by the gastrointestinal tract (GIT) lumen, intestines, and intestinal microbes, especially when taken orally [22]. β-glucosidase is an enzyme present in the gut that releases glucose by hydrolyzing the glycosidic bond between the sugar and benzyl groups. Death due to cyanide toxicity is caused by cyanide interference in mitochondrial oxygen utilization, leading to cellular hypoxia and lactic acidosis. This is because it binds to the ferric ion present on cytochrome oxidase, inhibiting electron transport and oxidative metabolism [23,24].

**Figure 2:**
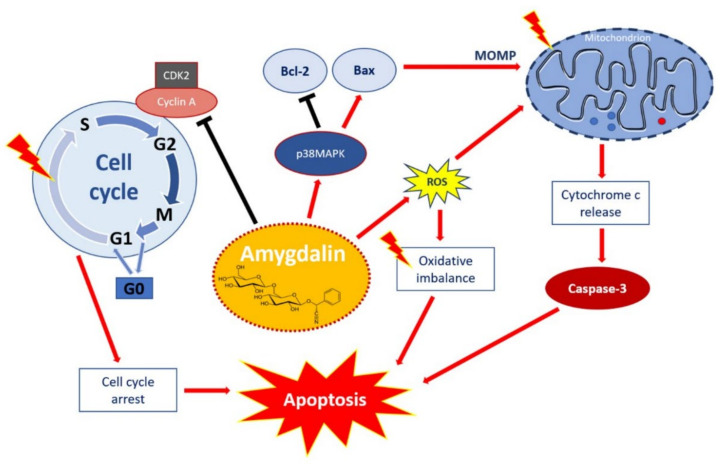
Apoptotic effect of amygdalin via multiple cellular signaling pathways. Amygdalin activates p38MAPK, which affects the death stimuli, activates Bax apoptotic proteins, and inhibits Bcl-2 antiapoptotic proteins. Apoptosis-related proteins induce mitochondrial outer membrane permeabilization (MOMP), a decisive event in the process of cytochrome c release. Activation of the release of cytochrome c as a mitochondrial response to proapoptotic stimuli via the mitochondrial or intrinsic apoptotic pathway ultimately leads to the activation of caspases, including caspase-3, which induces apoptosis. Amygdalin induces ROS overproduction, which compromises the oxidative balance and eventually leads to apoptosis. Amygdalin downregulates cyclin-dependent kinase 2 (CDK2) and cyclin A, which induces cell cycle arrest in the G0/G1 phases. Amygdalin also inhibits cell transfer from the G1 to S phase, resulting in the inhibition of cell proliferation and growth. “Activation” is shown by red arrows and ‘inhibition’ shown by black arrows. Taken from *Pharmaceuticals* with permission of authors [51].

The final products of amygdalin enzymatic hydrolyzation—benzaldehyde together with hydrogen cyanide—are responsible for the bitter aroma and flavor of amygdalin in fruit and result in said amygdalin toxicity and also affect the activity of pepsin when taken orally [25,26]. However, the cytotoxicity results of Choi and his team [27] showed that amygdalin alone was not toxic after incubation for 24 h but showed cytotoxicity in terms of the inhibition of cell proliferation, reactive oxygen species (ROS) generation, and induction of apoptosis (Figure 2) only after β-glucosidase treatment. Thus, amygdalin poses a high risk, especially when administered orally [28], because cyanide is released in much larger amounts after oral ingestion than by intravenous administration, not only due to the rapid action of the intestinal microflora [29] but also due to chewing or grinding [30]. Plants containing amygdalin also include separated β-glycosidases, which mix when chewed [31].

Shim and Kwon [32] observed the metabolism of orally administered amygdalin in a simulated gastrointestinal cell culture and found that it degraded first to prunazine and later to mandelonitrile by β-glucosidase, then hydroxylated to hydroxymandelonitrile in the small intestine. No cyanide or benzaldehyde was released at this stage, indicating that cyanide is likely to be produced in the lower intestine, which is rich in bacterial microflora. Firmicutes, Bacteroidetes, and Actinobacteria are the main groups of bacteria that contribute to the release of cyanide in the gut. Lethal oral doses (LD50) for cyanide are 2.13–6 mg.kg^−1^ body weight, and it has been confirmed that 59 mg of cyanide is released from 1 g of amygdalin [24,33]. However, based on the available peer-reviewed reports from Makarevic et al. [34], highly purified amygdalin applied at “therapeutic” concentrations (orally 0.6~1 g.kg^−1^) is unlikely to cause toxicity [18,35]. Rather, cyanide poisoning is associated with an overdose, the further ingestion of apricot kernels, or additional intake of vitamin C in megadoses, which is known to increase the in vitro conversion of amygdalin to cyanide. Sahin et al. (2011) and Sauer at el. (2015) reported that, in children aged 2 and 4, 500 mg of amygdalin caused vomiting, apathy, diarrhea, accelerated breathing, and a blood cyanide concentration of 163 µg/L [26,36]. In an adult woman, 9 g of amygdalin caused vomiting, dizziness, and a blood cyanide concentration of 143 µg/L [37].

#### Treatment of Cyanide Toxicity

The history of the excessive ingestion of amygdalin has manifested itself many times in hypotension, paralysis, coma, and even fatal cyanide poisoning, although, in the plant kingdom, cyanogenic glycosides have a helpful defensive function. In the long term, the intake of such substances is often accompanied by anxiety, headache, dizziness, or even confusion [37,38].

Rhodanese is an enzyme present in mammals in the mitochondria of the liver, whose main function is to act in the process of converting highly toxic cyanide to less toxic thiocyanate in an optimal pH = 8 environment. Afterward, it manifests its so-called “Ping-pong” mechanism of action, in which rhodanese subsequently releases a water-soluble thiocyanate compound excreted through the kidneys out of the body through the urine from a competent substrate by the attachment of a sulfur atom upon the addition of a cyanide (CN) group. However, ascorbic acid (vitamin C), iodine, alloxan, hydrogen peroxide, or mercaptans can inhibit this activity [37,38,39].

In the event of cyanide poisoning, an arterial blood gas analysis should be performed for a rapid and effective diagnosis, followed by appropriate treatment [40]. Common is the administration of Cyanokit, which is hydroxocobalamin that does not cause clinically significant side effects, apart from chromaturia and a reddening of the skin. Cobalamin, or vitamin B12, is used for this purpose due to its ability to chelate a toxic cyanide compound due to its higher binding activity alone or in combination with thiosulphates, triggering the above-mentioned rhodanese pathway. Vitamin B12 can also be applied for detoxification with the formation of cyanohydrin, endogenous α-ketoglutarate, which is a nitrogen scavenger, and a glutamate and glutamine source that promotes protein synthesis and prevents protein degradation in muscles that can detoxify cyanide to form α-ketoglutarate cyanohydrin [41]. The probiotics have been debated so far, as they help to downregulate β-glucosidase-producing bacterial strains that cleave amygdalin to cyanide, but this method has not been sufficiently explored [22,42,43,44,45,46,47].

## 3. Elementary Processes in Inflammation

The diseases are very diverse. They do not have a single cause, nor do they affect a single organ or cell type. This diversity has given rise to inflammation as a defensive reaction that is “suitable” for the repair of various disorders, and therefore, inflammation is activated by very different disorders and injuries. Sometimes, the body engages the only part of this universal defense response. Its overactivation or abnormal regulation also damages its cells and systems. Originally, this process was also considered mainly a harmful reaction. However, in recent years, the basic molecular and cellular mechanisms involved in inflammation, as well as their interactions, have been clarified [52,53].

Nowadays, the function of inflammation is generally defined as the multicellular vascularized organisms’ protective response to stimulation by the attack of pathogens or endogenous signals, such as damaged cells, leading to the removal of the original cause of injury, removal of necrotic cells, and tissue repair [54]. Chronic inflammation has been linked to heart disease, diabetes, cancer, arthritis, and bowel diseases like Crohn’s disease and ulcerative colitis, according to the data analysis [55]. Leprosy, asthma, emphysema, bronchitis, vitiligo, and colorectal cancer have all been treated with amygdalin [33,56].

Inflammation is a key aspect of the tissue responses to harmful inflammatory substances. However, as part of this defense process, very effective toxic substances are also formed, which, although they are the basis of the defense against foreign harmful forms, can also damage their surrounding structures. Therefore, their formation is not only under strict regulatory control but, with the onset of the inflammatory reaction, anti-inflammatory mechanisms are activated, which reduces the risk of damage to their tissues [53].

This complex response involves leukocytes such as macrophages, neutrophils, and lymphocytes, also known as inflammatory cells. In response to the inflammatory process, these cells release specialized substances that include vasoactive amines and peptides, eicosanoids, pro-inflammatory cytokines, and acute-phase proteins, which mediate the inflammatory process by preventing further tissue damage and, ultimately, leading to healing and restoration of the function [57].

Arachidonic acid (AA), a polyunsaturated fatty acid covalently bound in esterified form, is present in most cell membranes of the body [3] and released by cytosolic phospholipase A_2α_ (cPLA_2α_). cPLA_2α_ is ubiquitous-specific and constitutively expressed in two cells and tissues, with its activation initiated by submicromolar calcium concentrations and phosphorylation [58]. After cleavage from the cell membrane with this solution, arachidonic acid is metabolized by two main pathways: cyclooxygenase (COX-1, COX-2, and COX-3), which leads to the formation of prostanoids-prostaglandins, prostacyclins, and thromboxanes, and the lipoxygenase pathway (LOX), which leads to the formation of leukotrienes and lipoxins [2,53]. Cyclooxygenases (COX) 1, 2, and 3 represent specific isoforms, although there are significant differences in their distribution in the body and physiological and pathophysiological activities. COX-3 is the latest discovered, and its exact functional meaning has not yet been fully elucidated. COX-1 and COX-2 show approximately 60% sequential identity [59], consisting of a long narrow lipophilic channel with a hairpin at its end [60]. However, COX-1, present in low concentrations in all the cells and constitutively dependent on circulating hormones, produces prostaglandins, providing certain homeostatic functions, such as maintaining normal gastric mucosa, affecting the renal blood flow, and reducing smooth muscle cell proliferation, thus significantly contributing to myocardial protection [61,62,63]. In contrast, the COX-2 enzyme, as an essential component of the inflammatory cascade, is closely involved in the production of prostaglandins that mediate pain and inflammation by increasing vascular permeability [64], allowing the extravasation of pro-inflammatory cells, proteins, and enzymes that mediate the reactions leading to edema [65]. Prostaglandins also sensitize pain fibers to mechanical and chemical stimuli [66]. Concentrations of COX-2, as well as its metabolites in macrophages, monocytes, endothelial cells, and fibroblasts, can be increased up to 20-fold during inflammatory stimuli due to the action of the proinflammatory cytokines tumor necrosis factor-alpha (TNF-α) and interleukin (IL-1 and IL-6), where different transcriptional factors lead to nuclear signal transduction [61].

### 3.1. COX-1 and COX-2 Pathways

Cyclooxygenase or prostaglandin H synthase (PGH) is a bifunctional enzyme with cyclooxygenase and peroxidase activity. It cyclizes by inserting two oxygen molecules AA to form cyclic prostaglandin G_2_ (PGG_2_), which is subsequently converted by the peroxidase reaction to hydroxyendoperoxide prostaglandin H_2_ (PGH_2_) [67]. It gives formation by specific enzymatic reactions of cells not only to prostaglandins D_2_, E_2,_ and F_2-α_ but, also, to prostacyclin (PGI_2_) and thromboxane (TXA_2_), which act as receptor-dependent mediators and intracellular secondary messengers [68]. A COX deficiency leads to nonequilibrium concentrations of these eicosanoids, bringing favorable and unfavorable physiological conditions to the human body [69,70], so it is important to investigate the pathway to understand such effects of COX inhibition.

The first isoform of COX-COX-1 was first purified from bovine vesicular glands in 1976 by a scientific team led by Miyamoto [71], and subsequently, in 1989, Simmons et al. detected COX-2 from chicken embryo fibroblasts [72]. Various scientific studies suggest that the two isoforms are similar in structure but function as different enzymes present in the endoplasmic reticulum—in the case of COX-1 [73] and the perinuclear envelope-COX-2 [74]. In a study by Feletou, Huang, and Vanhoutte [75], they compared these two pathways, noting that COX-1 enzymatically processes AA, but COX-2 uses 2-arachidonyl glycerol as a substrate in addition to AA, resulting in the second-mentioned products on which the synthesis of COX-1 cannot work. Therefore, the availability of precursors and the kinetics of these enzymes are essential factors determining the regulation of individual reactions [76].

#### Selectivity between COX-1 and COX-2 and the Action of Nonselective Drugs

Specifically, COX-1 and COX-2 are homodimers consisting of three domains: an epidermal growth factor-like domain, a membrane-binding domain, and a catalytic domain, that contains both a COX and peroxidase active site [77]. These two enzymatic isoforms differ in the 120th amino acid. Where COX-1 has isoleucine, COX-2 uses a smaller valine as a substitute, creating a smaller side folder to which many drugs bind as part of its selective action in the inflammatory process [60].

The nonselective action of nonsteroidal anti-inflammatory drugs (NSAIDs) has a common goal in the inhibition of cyclooxygenase without distinction when the necessary symptomatic manifestations of inflammation (pain, swelling, and fever) are averted, but some adverse side effects occur. Thus, prostaglandins in the gastrointestinal tract can maintain the physiological responses of hydrophobic air secretion to epithelial surface cells or the maintenance of the epithelial cell membrane [78]. Therefore, after the discovery of two isoforms of this enzyme, it was time to test the drugs with a selective effect, hoping to avert similar negative side effects.

However, some studies, e.g., Aid and Bosetti [59], presented strong evidence of the negative effects of the selective inhibition of both COX-1 and COX-2. COX-2 has been detected in the cerebral cortex, hippocampus, and amygdala with both neuronal and vascular associations and is known to utilize spatial learning in vivo. Traditional anti-inflammatory drugs acting on its pathway have been shown by their inhibitory effect to increase the risk of serious cardiovascular side effects, an increase in atherothrombotic events, as a result of which, even such selective COX-2 inhibitors were later withdrawn from the pharmaceutical market [79,80].

Finally, such adverse side effects led to the search for other therapies, therapies based on gene or cytokine therapy, as well as research into other natural anti-inflammatory drugs that may be an alternative to available drugs, such as the subject of this review—cyanogenic glycoside amygdalin.

## 4. Inflammation Mediators

The adaptive inflammatory response to stimuli of exogenous or endogenous origin (microbes, allergens, stressed, dysfunctional, dead cells, etc.) represents a whole network of cellular and molecular interactions [57], which can be divided into four stages: inducers, sensors, mediators, and effectors [81]. It is a process in which inductive inflammations, after their recognition by sensors (specific transmembrane pattern recognition receptors—PRR) [82], trigger the formation of many inflammatory mediators, which subsequently changes the functionality of the host tissue and organs, especially the vasculature and mobilization of the effectors or change in the physical position of white blood cells [83]. Their scope is not always only local; they can also act in more distant places [40]. These soluble molecules are secreted by neutrophils, macrophages, basophils, eosinophils, mast cells, helper and cytotoxic T-lymphocytes, as well as platelets and endothelial cells [84].

The biochemical aspect of diffusible inflammatory mediators allows the following division [53]:

1. Proinflammatory cytokines (interleukins and tumor necrosis factors)—glycoproteins mobilizing leukocytes and endothelial cells provide an acute-phase response.
2. Eicosanoids—lipid metabolites of arachidonic acid.
3. Chemokines—the stimulation of leukocyte chemotaxis.
4. Vasoactive amines (histamine and serotonin)—the cause of vasoconstriction/vasodilation that may increase vascular permeability.
5. Vasoactive peptides (bradykinin, kallikrein, and kinin)—an increase in the vascular permeability, pain, and stimulation of arachidonic acid metabolism.
6. Proteolytic enzymes (elastase and matrix metalloproteinases)—cooperation in the killing of phagocytosed microbial parts and the recruitment of white blood cells.
7. Parts of the complement (C3a and C5a)—activation of the mast cells, basophils, and platelets; they are chemotactically and anaphylatoxically active.

There is ample evidence to suggest that certain groups of cytokines are involved not only in the initiation but, also, in the persistence of pathological pain by the direct activation of nociceptive sensory neurons, as well as in the central sensitization caused by nerve injury or inflammation, thus contributing to the etiology of various pathological pain conditions [85,86]. Since the hitherto known mechanism of the anti-inflammatory effect of amygdalin is to selectively inhibit the arachidonic acid metabolites, as well as to block the transcription of the cytokines, we will take a closer look at them.

### 4.1. Inflammation Mediators: Eicosanoids Metabolites of Arachidonic Acid Pathway

These lipid mediators are formed from 20 carbon fatty acids by cyclooxygenase and lipoxygenase substances, with arachidonic acid being the most common substrate due to its presence at the place—phospholipid membrane inflammatory cells—in major higher amounts than the other potentially suitable polyunsaturated fatty acids [87,88]. However, some studies pointed to a reduction in the production of eicosanoids based on arachidonic acid when ingesting fish oil containing eicosapentaenoic acid (EPA), which may partially replace AA in inflammatory cell membranes [89].

Arachidonic acid eicosanoids collectively represent a category of prostaglandins, thromboxanes, leukotrienes, and lipoxins, which are important mediators of inflammation, various immune processes, even cancer, and chronic tissue remodeling. Although, on the other hand, their effects represent such biological activities as maintaining tissue homeostasis, smooth muscle contraction, or platelet aggregation [90]. This largest group of tissue hormones has a variety of modified structures and functions, based on the sample (mono or di); position (5, 8, 12, or 15); and stereospecific (R/S) of oxygen incorporation into the substrate base. The type of receptor expressed on the respective cell surface is also crucial [91]. In the normal state of cells, eicosanoids are formed in small concentrations, and the subsequent upregulation occurs mainly after inflammatory stimulation [92]. This increases the expression of microsomal prostaglandin E synthase-1 and COX-2, which accelerate the biosynthesis of prostaglandins and thromboxane from arachidonic acid [93,94]. Although eicosanoids have a short half-life, they contribute to many biological activities in a paracrine or autocrine manner. They play a crucial dual role in regulating the innate immunity and eliminating inflammation through the seven transmembrane domains to G protein-coupled receptors found on other cells. Eicosanoid receptors control the release of secondary messengers such as cyclic adenosine monophosphate (cAMP), diacylglycerol, and inositol-1,4,5-triphosphate, which mediate many of their cellular effects. The first of these roles is when tissues are inflamed or infected; in addition to pain and swelling, AA metabolites increase inflammatory signals to mobilize leukocytes, immune cells to help with resistance and eliminate pathogens. This also produces a cytokine storm. In the second task, eicosanoids can balance the induced inflammatory signals by producing cleaving metabolites that act as host protections, because, as already mentioned, if inflammation is not adequately controlled, it has a very negative effect [95]. Eicosanoids include, in addition to arachidonic acid metabolites, hydroxyeicosatetraenoic acids, hydroxyeicosapentaenoic acids, eoxins, isoprostanes, and resolvins [96,97].

#### 4.1.1. Prostaglandins

Prostaglandins are a family of hormone-like molecules produced by all cell types. They maintain homeostatic functions and mediate pathogenic mechanisms, including the inflammatory response. They are involved in preserving the inflammatory process by increasing vascular permeability and enhancing the effect of other inflammatory mediators, such as kinin, serotonin, and histamine [98], thereby contributing to redness, increased blood flow, and plasma efflux in the area of acute inflammation, leading to edema [57]. The major bioactive prostaglandins (PG) generated in vivo are PGE_2_, PGI_2_, PGD_2_, and PGF_2α_, mentioned above.

- PGE_2_: This bioactive lipid elicits a wide range of biological effects, from cell proliferation, apoptosis, angiogenesis, inflammation, and cancer to immune surveillance [99]. However, its role in inflammatory conditions is not clear. This prostaglandin acts on neurons in the hypothalamic thermoregulatory network and causes an increase in body temperature and may promote the activation of inflammatory Th17 cells but, on the other hand, inhibits the production of IL-2 and IL-12 in other subsets of T cells. This difference appears to be due to the dependence on the expression of different receptors, as well as the cell type [100]. It is also known to regulate the activation, maturation, migration, and secretion of cytokines of several immune cells, primarily those involved in innate immunity—namely, macrophages, neutrophils, natural killing cells, and dendritic cells. PGE_2_ synthesis is increased by the expression of one of the cyclooxygenases [101].
- PGI_2_: Prostacyclin or PGI_2_ is the end product of AA metabolism first observed in 1976 with the participation of Moncada [102]. PGI_2_ has also been found to be essential for maintaining cardiovascular health because it inhibits platelet aggregation and has strong vasodilatory effects through smooth muscle relaxation [103]. By activating prostacyclin receptors (IP receptors), PGI_2_ has a positive cardiovascular effect, e.g., to inhibit vascular smooth muscle cell proliferation. However, these receptors are nonfunctional under pathological conditions and, thus, have deleterious effects that are in contrast to their physiological protective effects via thromboxane-prostanoid (TP) receptors [104]. In addition to various immunomodulatory effects on T cells, PGI_2_ also regulates B-cell functions [105]. It is involved in the transmission of pain sensation, mediates vascular permeability during the inflammatory process, and regulates the rate of vascular glomerular filtration. In the state of inflammation, the PGI_2_ signal promotes inflammatory pain [106]. The presence of receptors for this lipid mediator has also been found in the central nervous system [107]. The anti-inflammatory effects of prostacyclin are largely controlled by cAMP (cyclic adenosine monophosphate) and suppression of the NF-κB inflammatory signaling cascade [108].
- PGD_2_: PGD_2_ is an important prostanoid that is released primarily from mast cells but, also, from other immune cells, such as TH_2_ and dendritic cells. PGD_2_ performs its biological functions mainly through two G-protein coupled receptors, PGD_2_ receptors 1 (DP1) and 2 (DP2). The effect of DP1 is to inhibit platelet aggregation, vasorelaxation, bronchodilation, increased mucus production, and airway hyperresponsiveness. Through interaction with the latter, PGD_2_ stimulates the significant chemotaxis and degranulation of inflammatory cells [109]. PGD_2_ mediates several physiological effects in various tissues and organs, in addition to those mentioned; its functions include the induction of sleep or the secretion of mucus in the airways [110]. It is also a powerful inflammatory stimulant, but the time perspective plays an important role if it acts in the early or late phase of inflammation [111]. This prostanoid also acts gastroprotective by reducing acid secretion from parietal cells while increasing the blood flow and stimulating mucus secretion [112]. The presence of an allergen triggers the rapid production of PGD_2_ in sensitized individuals, e.g., in patients with asthma, where concentrations of this biologically active substance can increase up to 150-fold than before the allergen. Its amount also increases in allergic rhinitis and atopic dermatitis. So far, several studies are known to indicate that mast cells are the main source of prostanoid PGD_2_ at sites of allergy-associated inflammation [113]. It increases the migration, activation, and survival of leukocytes in various disorders associated with allergies. At the periphery, hematopoietic PGD synthase (hPGDS) acts on the arachidonic acid and COX pathways by isomerizing PGH_2_ to PGD_2_, which scientists seek to use in therapeutic procedures for allergic inflammation and to develop suitable hPGDS inhibitors [111].
- PGF_2α_: PGF_2α_ is a key signaling mediator in childbirth because it is involved not only in stimulating uterine contraction but, also, in mediating uterine transition by regulating the expression of uterine activating proteins and amplifying proinflammatory cytokine and chemokine production [114]. PGF_2α_ increases the intracellular calcium concentration by stimulating the release of stored calcium, leading to a phase contraction that allows blood to flow to the fetus between contractions and optimizes the ability of the uterus to expel the fetus [115]. It has also been shown that the concentration of PGF_2α_ in maternal plasma is higher before childbirth compared to the first stage of labor, and there is no significant change even at the time of childbirth, which may mean that PGF_2α_ has other roles during this period [116]. This prostaglandin can affect the luteal cell viability either by inducing proliferation or cell death through apoptosis or necrosis, depending on its local and systemic effects [117]. PGF_2α_ has shown a positive effect on increasing decidual gelatinolytic activity by increasing matrix metalloproteinase (MMP)-2 and MMP-9 while decreasing the tissue inhibitor of MMP expression (TIMP) -1 [118]. PGF_2α_ as a bone resorption agent also regulates the expression of fibroblast growth factor-2 and fibroblast growth factor receptor (FGFR) in osteoblasts [119].

#### 4.1.2. Thromboxanes

Thromboxanes are eicosanoids derived from arachidonic acid and are related to prostaglandins. They represent substances produced by platelets causing blood clotting, narrowing of blood vessels, and having a positive effect on platelet aggregation. Thromboxane A_2_ (TXA_2_) is active for a very short time and is converted by hydrolysis to an inactive form—thromboxane B_2_ (TXB_2_) [120].

- TXA_2_: It is an unstable metabolite AA with a half-life of about 30 s, is synthesized from prostaglandin H_2_ by thromboxane synthase, and is nonenzymatically degraded to biologically inactive TXB_2_ [121,122]. TXA_2_ is formed mainly in platelets (which express only COX-1), with its production increasing during their activation and thromboxane acting through its thromboxane receptor (TP)α receptor. It promotes platelet aggregation [123], vasoconstriction, smooth muscle proliferation, and the activation of endothelial inflammatory responses [124]. It is part of an essential repair mechanism for wound healing, including damaged vessel walls, and is responsible for early tissue regeneration through TP receptor signaling [125]. The thromboxane metabolite 12-Hydroxyheptadecatrenoic acid (12-HHT) is particularly important for skin regeneration [126]. TXA_2_ was originally described as being released from platelets but is known to be released by several other cells, including macrophages, neutrophils, monocytes, and endothelial cells [121].

#### 4.1.3. Leukotrienes

Leukotrienes (LT)—namely, LTB_4_, LTC_4_, LTD_4_, and LTE_4_—are important biologically active lipid substances derived from AA via another 5-lipoxygenase [127]. They are potent biological mediators in the pathophysiology of inflammatory diseases and are synthesized by a variety of cells, including mast cells, eosinophils, basophils, and macrophages. They trigger contractile and inflammatory processes through specific interactions with cell surface receptors that belong to the G protein-coupled receptor superfamily [128].

- LTB_4_: Leukotriene B_4_ is one of the most effective chemoattractant mediators of inflammation. It stimulates neutrophil chemotaxis, chemokinesis, and adhesion to endothelial cells and activates neutrophils, leading to the release of enzymes, mediators, and degranulation [129]. It activates them especially for the production of reactive oxygen species and the release of lysosomal enzymes. LTB_4_ is also involved in inflammatory pain by lowering the nociceptive threshold through neutrophil-dependent processes [130]. High concentrations of this leukotriene are likely to be found in inflammatory secretions in patients with arthritis and cystic fibrosis. Its synthesis is inhibited by colchicine, an anti-inflammatory agent effective in the treatment of gout [53].
- LTC_4_: LTC_4_, together with LTD_4_ and LTE_4_, are considered known cysteinyl leukotrienes (CysLT) that accompany asthmatic conditions [131]. CysLTs act on vasoconstriction and increase the vascular permeability, allowing plasma macromolecules to be eliminated, leading to airway edema, which characterizes asthma. In addition, CysLT stimulates mucus secretion and inhibits mucociliary clearance. They are also characterized as active ingredients of “slow-reacting substances of anaphylaxis” [132]. LTC_4_ formation occurs by the conjugation of LTA_4_ and reduced glutathione, with eosinophils, basophils, mast cells, macrophages, and platelet-adherent granulocytes being the primary sources [133]. The cysteinyl leukotriene has been implicated in the induction of both oxidative stress and apoptosis [134] and is a potent bronchoconstrictor [135].
- LTD_4_ and LTE_4_: LTD_4_ is a member of the cysteinyl class, and its effects are mediated mainly by the leukotriene receptors CysLT1 and CysLT2. It has been implicated in a variety of inflammatory effects, including bronchoconstriction, vasoconstriction, increased postcapillary permeability with swelling, and smooth muscle contraction [136]. There is evidence that LTD_4_ and LTE_4_ are potent and specific chemoattractants for eosinophils that appear to be important in the pathophysiology of asthma [137].

#### 4.1.4. Lipoxins

This group of endogenous anti-inflammatory mediators supports the resolution [138]. They are key players in reducing excessive tissue damage and chronic inflammation and regulate components of both the innate and adaptive immune systems, including neutrophils, macrophages, and T- and B-cells [139]. Lipoxins (LX) also control the production of other eicosanoids by a mechanism of the stimulation of prostaglandin production [140]. They can modulate the concentrations of various transcription factors, such as NF-κB or activator protein-1, and others to control the expression of multiple inflammatory genes [141].

- LXA_4_: It is an endogenous eicosanoid mediator, which has not only a dual pro-resolution ability but also has anti-inflammatory properties. It is an inducer of prompt arterial dilatation. It suppresses leukocyte-mediated damage and promotes monocyte chemotaxis and the phagocytosis of apoptotic neutrophils [142], as well as the phlogistic uptake of apoptotic polymorphonuclear neutrophils [143].
- LXB_4_: LXB_4_ has a different structure than the rest of the lipoxin family, and its signaling pathway is different from LXA_4_. It is a neuroprotective mediator that acts locally to suppress inflammation by inhibiting the production of the T-cell-activated cytokine TNF-α. Its production is regulated by the nucleotide-binding domain (NOD)-like receptor protein 3 (NLRP3) inflammasome activity [144]. It also has strong effects on macrophages and noninflammatory monocyte activation [138]. It can stimulate Immunoglobulin G (IgG) secretion in memory B cells, and the study by Kim et al. [145] also provided evidence of its ability to increase IgG-secreting B cells.

### 4.2. Effect of Amygdalin on the COX-2 Pathway

The study by Yang and his team [146] provided clear evidence of this effect, with amygdalin inhibiting lipopolysaccharide (LPS)-stimulated increases in COX-1 messenger Ribonucleic acid (mRNA) expression, COX-2 mRNA expression, and PGE_2_ synthesis in murine BV2 microglial cells. COX-2 mRNA expression was more efficiently downregulated by this glycoside compared to COX-1 mRNA expression. A research team led by Kunga [147] highlighted the ability of amygdalin to inhibit, in a dose-dependent manner, TNF-α expression, inducible nitric oxide synthase, and COX-2, as well as NF-κB protein expression, and increased the levels of oxidative stress-related proteins, e.g., nuclear factor-erythroid-2-related factor 2 (Nrf2). This is because COX-2 activity is associated with nitric oxide (NO) radicals formed based on NO synthase activity, and, as mentioned, amygdalin inhibits it, thus indirectly suppressing the production of PGE_2_, the main metabolite of COX-2 [148]. The results of Tang et al. [149] suggest that amygdalin treatment dramatically inhibited TNF-α, IL-6, and IL-1β production, as well as LPS/D-galactosamine (GalN)-induced iNOS and COX-2 protein expression in mice, thus ameliorating acute liver damage (ALI). The results of Gago-López et al. (2021) confirmed the expression of important mediators of psoriasis initiation and epidermal hyperplasia, such as TNF-α [150]. According to Wang et al. (2020), amygdalin, the main component of Prunus persica (L.) Stokes, decreased inflammatory cytokines (Il-6, Tnf-α) in a mouse model [150].

## 5. Inflammation Mediators: Cytokines

Cytokines are small proteins released by cells that have a significant specific effect on interactions, communication between cells, and they interfere with the inflammatory response to an extremely wide extent, especially at picomolar, sometimes nanomolar, concentrations. They act mostly locally in an autocrine or paracrine manner, but when they escape into the blood or lymphatic circulation, they can affect the entire system [151].

Cytokines include interleukins, tumor necrosis factors, lymphokines, monokines, tumor necrosis factors, and interferons but, also, colony-stimulating factors, transforming factors, peptide growth factors, heat shock proteins, and glucose-regulated proteins [53].

The cytokine system is an important and effective mechanism of homeostasis when its activation takes place locally and the individual cytokines act in the immediate vicinity, whether in diffusible form or bound to cell surfaces. However, if the production of cytokines is long-lasting and constantly maintained or is systemic, in such a situation, the cytokines exacerbate inflammatory, autoimmune, and malignant conditions [152].

In the process of inflammation, the main active cytokines are TNF, IL-1, and IL-6, which are produced by resident cells such as macrophages, mast cells, and lymphocytes, and mediate cell migration towards the affected site, fever (streamlining metabolic processes, etc.), edema, and, also, tissue sensitization to pain. In particular, TNF-α and IL-1β are mainly secreted to release bioactive lipid mediators—prostaglandins and sympathomimetic amines [86].

### 5.1. Regulation of Cytokines

Proinflammatory cytokines are key players responsible not only for the subsequent course of innate immune responses but, also, for the development of adaptive immunity in this direction, which stimulates them to differentiate and activate specific T-cell subgroups. Insufficient regulation or predominance of the factor causing destabilization of the body can result in harmful inflammation like rheumatoid arthritis [153]. In particular, the actual presence of cytokines at the site of inflammation plays a role in tissue destruction. It is a long-term process, which is accompanied by attempts of the body to recover. Such a disbalance can also cure chronic inflammatory conditions or autoimmune diseases [154].

The mechanism of cytokine regulation is based on two pillars: the first is receptor antagonism and the second is the contrast of their effects, e.g., certain groups of cytokines stimulate the production of destructive matrix metalloproteases while another group promotes the production of tissue inhibitors of matrix metalloproteases, thus controlling the balance between these effects [155].

The latter mechanism is of key importance during inflammatory processes and is used as a therapeutic approach, e.g., IL-1 genes encode IL-1α and IL-1β and, also, the antagonist receptor (IL-1ra), where the state of homeostasis between IL-1, IL-1ra, and other natural inhibitors is maintained by their equilibrium states [156]. Blocking ligands-soluble receptors, e.g., IL-1sR and TNF-sR—can also act [53,157].

### 5.2. Effect of Amygdalin on Inflammation-Suppression of Proinflammatory Cytokine Release

IL-1α: More than any other, the IL-1 family of cytokine ligands and receptors is primarily associated with acute and chronic inflammation [158], so basic inflammatory reactions such as the induction of cyclooxygenase type 2, production of multiple cytokines and chemokines, and increased expression of adhesion molecules or nitric oxide synthesis are indistinguishable responses to IL-1 ligands [159]. The IL-1α signaling pathway leads to the activation of NF-κB and activator protein (AP-1) in the cytosol [160]. Studies suggest that amygdalin has an anti-inflammatory effect due to the reduced expression of the proinflammatory cytokine IL-1α [161]. Histological evaluation of the burn wounds in this study showed a remarkable improvement in earlier healing, a smaller wound area, and a better scar in the amygdalin-treated group, which also showed a significant decrease in inflammatory cytokine concentrations.

IL-1β: IL-1β is a pleiotropic cytokine that can mediate increased procoagulant activity and permeability in endothelial tissue during inflammatory conditions [162]. IL-1β is involved in vascular inflammation, hemodynamics, and angiogenesis [163]. Upon completion of the maturation process of this interleukin occurring in the cellular protein structure inflammasome, it is involved in the regulation of immune processes through the activation of the transcription factor NF-κB and mitogen-activated protein kinase (MAPK)38 [164,165]. Scientists Hwang et al. [166] and many others have demonstrated the anti-inflammatory ability of amygdalin. Hwang et al. demonstrated it in an in vivo study on rats in which they induced inflammation intramuscularly with carrageenan and monitored the molecular markers of inflammation and pain with the addition of amygdalin at concentrations of 0.005, 0.05, and 0.1 mg.kg^−1^. At the lowest selected concentration of amygdalin (AMG), there was a significant inhibition of the expression of IL-1β, TNF-α, and c-Fos protein as a marker of neuronal activity. Inhibition of the expression of this cytokine by amygdalin in a dose-independent manner was also demonstrated by studies by Luo et al. [10] in collagen type II-induced arthritis in rats.

IL-6: IL-6 represents a strategic bridge that connects the innate and adaptive immune systems by attracting monocytes and lymphocytes to replace neutrophils in the transition from innate to adaptive immunity [167,168]. It is a pleiotropic cytokine formed by macrophages and neutrophils but also endothelial and epithelial cells and others. IL-6 has also been shown to induce PGE_2_, which can inhibit the synthesis of the proinflammatory mediators TNF-α and IL-1 [169]. It also induces the production of acute-phase proteins in liver cells. IL-6 transmits signals through its unique receptor system. It interacts with a cell surface type I receptor complex that consists of a ligand-binding glycoprotein called IL-6Rα (also called the cluster of differentiation (CD)126) and a signal transduction component of glycoprotein (GP) 130 (also called CD130) [170]. Zhang et al. [171] tested the anti-inflammatory effect of our natural substance in connection with IL-6 and IL-1β in search of a suitable treatment for patients with acute lung injury (ALI). This is because ALI is caused by direct or indirect damage to lung alveolar epithelial cells and endothelial cell capillaries, where LPS is responsible for activating the NF-κB signaling pathway. As a result of this activation, LPS indirectly affects the expression of cytokine genes (TNF-α, IL-1β, and IL-6), leading to lung tissue damage. In conclusion, the results obtained by Zhang et al. showed that amygdalin suppresses LPS-induced ALI by inhibiting the synthesis of the inflammatory cytokines IL-6 and IL-1β by affecting the NF-κB and NLRP3 signaling pathways. Gago-López et al. (2021) demonstrated that amygdalin analog treatment leads to the reduced expression of proinflammatory cytokines that are highly expressed in psoriasis patients, such as IL6 [172].

TNF-α: Tumor necrosis factor-alpha is a pleiotropic proinflammatory cytokine that is produced mainly by macrophages but also by several other cells, e.g., endothelial cells [173]. Its key functions include the induction of pro-inflammatory proteins (chemokines, cytokines, and adhesion molecules) through the activation of the transcription factor NF-κB [174]. In a study by Hu F., Hu Y., and Peng [175], amygdalin, in combination with atorvastatin, a statin drug used to prevent cardiovascular disease in high-risk individuals and to treat abnormal lipid levels, demonstrated significant changes in the outcomes and reduced the cytokine mRNA levels. TNF-α and IL-6 conversely increase the activities of the antioxidant enzymes glutathione peroxidase, superoxide dismutase, and catalase in rat models of endometriosis.

MCP-1: Monocyte chemoattractant protein-1 (MCP-1/ CCL2) is one of the key chemokines that regulate monocyte/macrophage migration and infiltration [176]. It induces monocyte activation, and recruitment can increase the expression of adhesion molecules and the production of cytokines by monocytes [177]. It can directly upregulate the proinflammatory cytokine IL-6 and the adhesion molecule intercellular adhesion molecule-1 (ICAM-1). Thus, MCP-1 is not only a chemokine but also a proinflammatory mediator, inducing the formation of proinflammatory molecules [178]. MCP-1 exerts its effects by binding to G-protein-coupled receptors on leukocyte surfaces, leading to Akt kinase activation and phosphatidylinositol triphosphate formation for the highest chemokine concentrations [179,180]. However, Lv and the research team [181], to find an appropriate course for the formation of atherosclerosis, registered a significant (*p* < 0.05) immunomodulatory effect of AMG on this factor, as well as on IL-6. MCP-1 concentrations were measured in atherosclerotic arteries. Amygdalin was injected into the abdomen of mice at doses of 1, 3, 10 mg.kg^−1^ for 4 weeks, where, at its mean indicated concentration, a Western blot analysis showed a significant reduction in MCP-1 protein production. The research of Zhao and Yang [182] presented similar evidence for AMG, the protective role of amygdalin in the development of atherosclerosis. Here, again, at a concentration of 10 mg.kg^−1^, it significantly (*p* < 0.05) reduced the MCP-1 protein expression compared to the AMG-untreated sample. These data suggest that the natural product under investigation could directly or indirectly inhibit the development of atherosclerosis and promote a more stable plaque phenotype by inhibiting the expression of several pro-inflammatory molecules, including MCP-1.

### 5.3. Basic Principles of Signaling Pathways in Which Amygdalin Is Involved

#### 5.3.1. Janus Kinase 2 and Signal Transducer and Activator of Transcription 3 (JAK2/STAT3) Pathway

This signaling pathway is involved in various inflammatory and anti-inflammatory signaling pathways and several physiological and pathological regulatory processes [183].

Signaling begins with the attachment of a ligand cytokine to a target cell receptor located on its membrane. Janus kinases (JAKs) are intracellularly localized receptor-associated enzymes that catalyze the transfer of a phosphate group from nucleoside triphosphates—mostly, Adenosine triphosphate (ATP)—to the amino acid tyrosine in proteins. The binding of a cytokine to a receptor results in their proximity in the intracellular space and mutual phosphorylation, thereby activating and phosphorylating the tyrosine residues of the receptor cytoplasm. The STAT (signal transducer and activator of transcription) protein present in the cytosol can then be bound to phosphotyrosines via its Src Homology 2 (SH2)2 domain. JAKs subsequently again exert their tyrosine kinase activity on the attached STAT proteins, leading to the dissociation of these proteins from the receptor or separation from phosphotyrosines. The SH2 domain mediates binding to the phosphotyrosine of the second STAT molecule, resulting in a homo/heterodimer translocated to the cell nucleus. Here, it initiates transcription and gene expression [180,183,184].

#### 5.3.2. NF-κB Pathway

NF-κB is a central mediator of proinflammatory gene induction and functions in both innate and adaptive immune cells [185]. NF-κB is not formed by a single protein but a complex of inducible transcription factors [186], which is the first to respond to harmful cellular stimuli, e.g., ROS [187] or ionizing radiation [188]. The activation of NF-κB is controlled by two distinct pathways, which have been termed canonical and noncanonical pathways [189]. In the case of the former, the NF-κB heterodimer composed of RelA and P50 proteins are maintained in an inactive state by cytoplasmic association with the inhibitory protein IKBα [186]. Activation of the canonical pathway by binding of a signaling molecule in the form of IL-1 or TNF-α/β to the TLR (Toll-like receptor) [190], TNFR (Tumor Necrotizing Factor Receptor) [180], or RANK (Receptor activator of NF-κB) [191] leads to Inhibitory Kappa B Kinase (IKK) (IκB kinase)-mediated phosphorylation, ubiquitination, and the subsequent proteasomal degradation of IκB (Inhibitor of NF-κB), which allows the transcription factor to translocate to nuclear cells and the activation of genes carrying related binding sites for cytokine production or cell survival [189]. TNFR (TNFR1 and TNFR2) are intracellularly associated with TRAF adapter proteins (TNF receptor associating factors) either directly or through other proteins—TRADD (TNF receptor-associated death domain), whose role is to regulate inflammatory and apoptotic pathways [180]. TRAF-2 in a complex with TRADD mobilizes the IAP protein (Inhibitor of apoptosis protein), which binds to TRAF-2, leading to activation of the transcription factor NF-κB by the RIP protein (Receptor-interacting protein kinases) [192], activating the already mentioned IKK kinase. Upon activation, NF-κB regulates the expression of nearly 400 different genes, which include the enzymes COX-2 and iNOS [190].

### 5.4. Molecular Mechanism of Amygdalin Action on Immune Function In Vitro

At the beginning of the 21st century, a scientific team led by Baroni [193] investigated the protective effects of amygdalin on the immune function of the vascularized organism. This glycoside then demonstrated the ability to increase polyhydroxyalkanoate (PHA)-induced T-cell proliferation in human peripheral blood, which subsequently secreted IL-2 and Interferon-gamma (IFNγ) to inhibit transforming growth factor (TGF)-β1 production. The TGF-β superfamily includes endogenous growth inhibitory proteins; increased TGF-β expression often correlates with malignancy in many cancers and a defect in the response of cell growth inhibition to TGF-β [194]. The dysregulation of immunosuppressive functions of this group is also involved in the pathogenesis of autoimmune diseases mediated by the presence of other cytokines [195].

In 2019, Tang and his team [149] observed that amygdalin treatment effectively improved histopathological changes in the liver by reducing the levels of malondialdehyde (MDA), myeloperoxidase (MPO), alanine aminaminase (ALT), and aspartate aminotransferase (AST). Amygdalin also downregulated the secretion of TNF-α, IL-1β, and IL-6, thereby inhibiting hepatic organ inflammation.

Data obtained from several research teams [149,161,171,182,196] suggest that the anti-inflammatory activity of the studied natural glycoside lies in the inhibition of the expression of several proinflammatory molecules, whereby regulating the NF-κB signaling pathway, NLRP3, as well as the Phosphatidylinositol 3-kinase/Protein Kinase B (PI3K/AKT) and JAK2/STAT3 pathways, prevents their transcription. Inflammatory stimuli are recognized by host cells through specific transmembrane receptors, where the interactions of these receptors with the appropriate immune systems lead to the transmission of signals to their nucleus, where a selective set of genes is activated through transcriptional and posttranscriptional mechanisms (MAPK (mitogen-activated protein kinase), NF-κB (nuclear factor kappa-light-chain-enhancer of activated B cells), JAK2/STAT3 (Janus kinase 2/Signal transducer and activator of transcription 3), etc.) [81,197,198,199]. Inflammatory responses are subsequently coordinated by the products of such genes—cytokines, chemokines, and interleukins. Amygdalin has shown this anti-inflammatory potential in inhibiting IL-1α, IL-1β, IL-6, IL-9, TNF-α, and MCP-1 [149,166,171,196,200,201,202]. Tang et al. [149] demonstrated in their research the inhibition of IL-6 expression by amygdalin, which, at a deep molecular level, prevented the translocation of NF-κB from the cytosol to the nucleus and, thus, prevented its transcription. Moreover, amygdalin ameliorates excessive oxidative stress, inflammation, and the renal tissue fibrosis of diabetic nephropathy mainly by suppressing the TGF-β1/Smad signaling pathway and regulating the key enzymes of ECM degradation [203].

## 6. Conclusions

Our immunity has defense mechanisms, such as inflammation. Amygdalin, as a bioactive product, tends to selectively act on arachidonic acid in the inflammatory process, but the molecular mechanism and specific circumstances of this effect in vitro and in vivo have not been fully elucidated. However, other published scientific studies to date have indicated that amygdalin has potential as a natural biologically active compound. We hope that this presented overview can be helpful to the research in the fields of molecular biology, toxicology, and medicine.

## Figures and Tables

**Table 1:** Amygdalin metabolites, adverse effects, treatment for overdose, and pharmacological function.

Metabolites	Benzaldehyde	[22,23]
	Mandelonitrile	[29]
	Prunazine	[29]
Adverse effects	Nausea and vomiting	[48]
	Headache	
	Dizziness	
	Cyanosis	
	Liver damage	
	Hypotension	
	Ptosis	
	Ataxic neuropathies	
	Fever	
	Mental confusion	
	Coma	
	Death	
Treatment for overdose	Hydroxocobalamin	[49]
Pharmacological function	Antitumor	[7,50]
	Antifibrotic	
	Anti-inflammatory	
	Analgesic	
	Immunomodulatory	
	Antiatherosclerosis	
	Ameliorating digestive system	
	Ameliorating reproductive system	
	Improving neurodegeneration	
	Improving myocardial hypertrophy	
	Reducing blood glucose	

## Data Availability

All data are provided in the manuscript.

## Sample Availability

Samples of the compounds are not available from the authors.
